# Voltage-Dependent Gating in a “Voltage Sensor-Less” Ion Channel

**DOI:** 10.1371/journal.pbio.1000315

**Published:** 2010-02-23

**Authors:** Harley T. Kurata, Markus Rapedius, Marc J. Kleinman, Thomas . Baukrowitz, Colin G. Nichols

**Affiliations:** 1 Department of Anesthesiology, Pharmacology, and Therapeutics, University of British Columbia, Vancouver, Canada; 2 Institute of Physiology II, Friedrich Schiller University, Jena, Germany; 3 Department of Cell Biology and Physiology, Washington University School of Medicine, St. Louis, Missouri, United States of America; 4 Center for Investigation of Membrane Excitability Disorders (CIMED), Washington University School of Medicine, St. Louis, Missouri, United States of America; University of Texas at Austin, United States of America

## Abstract

The voltage sensitivity of voltage-gated cation channels is primarily attributed to conformational changes of a four transmembrane segment voltage-sensing domain, conserved across many levels of biological complexity. We have identified a remarkable point mutation that confers significant voltage dependence to Kir6.2, a ligand-gated channel that lacks any canonical voltage-sensing domain. Similar to voltage-dependent Kv channels, the Kir6.2[L157E] mutant exhibits time-dependent activation upon membrane depolarization, resulting in an outwardly rectifying current-voltage relationship. This voltage dependence is convergent with the intrinsic ligand-dependent gating mechanisms of Kir6.2, since increasing the membrane PIP_2_ content saturates Po and eliminates voltage dependence, whereas voltage activation is more dramatic when channel Po is reduced by application of ATP or poly-lysine. These experiments thus demonstrate an inherent voltage dependence of gating in a “ligand-gated” K^+^ channel, and thereby provide a new view of voltage-dependent gating mechanisms in ion channels. Most interestingly, the voltage- and ligand-dependent gating of Kir6.2[L157E] is highly sensitive to intracellular [K^+^], indicating an interaction between ion permeation and gating. While these two key features of channel function are classically dealt with separately, the results provide a framework for understanding their interaction, which is likely to be a general, if latent, feature of the superfamily of cation channels.

## Abstract 2

An unusual mechanism of ion channel regulation generates voltage-dependent gating in the absence of a canonical voltage-sensing domain.

## Author Summary

Ion channels are proteins that regulate the transfer of ions across the cell membrane. The ions travel via a pore formed by the different subunits that constitute the channels, and this pore can be gated by changes in the electrical field across cell membranes. The canonical mechanism underlying voltage dependence of gating relies upon a widely conserved structural motif called the voltage sensor, which undergoes conformational changes when charged amino acids within the motif respond to voltage and consequently affect the opening of the ion channel pore. In the present study, we have identified a non-canonical mechanism that surprisingly generates voltage-dependent changes in the activity of a ligand-gated ion channel that has no voltage sensor. Our observations suggest that ions flowing through the ion channel pore can significantly affect channel activity, and we suggest that voltage-dependent changes in ion distribution in the “cavity site” of the channel can influence opening and closing of the channel independent of canonical voltage sensors.

## Introduction

While the entire complement of ion channels in a given cell contributes to the membrane voltage, only a subset (the voltage-gated cation channel family) responds significantly to changes in membrane voltage, and the molecular mechanisms underlying their voltage dependence remain the subject of considerable scrutiny [1]–[6]. Voltage-gated cation channels are typified by a modular 6-transmembrane segment (S1–S6) architecture, with the S5 and S6 helices forming a core pore-forming module, and the S1–S4 helices forming a canonical voltage-sensing domain (VSD) [7]. The VSD, and particularly a subset of positively charged amino acids in the S4 transmembrane segment, is essential for this voltage-dependent gating [8]–[10]. Inwardly rectifying potassium (Kir) channels possess a similar core K^+^-selective pore module but lack the VSD, and the gating mechanisms of this channel family are generally considered independent of voltage [11]–[13]. Instead, Kir channels are physiologically regulated by ligands specific to each channel subfamily, such as Gβγ subunits (Kir3 channels), protons (ROMK1 and others), or nucleotides (Kir6 channels) [14]–[16]. In addition, anionic “signaling” phospholipids such as PIP_2_ interact with the cytoplasmic domains of all known Kir channels and increase channel activity [17].

Despite clear distinctions at the level of primary sequence, predictions of functional behavior based on structural properties do not always hold firm. For example, CNG channels contain a VSD but exhibit little intrinsic voltage-dependent gating [18]. A similar lack of voltage dependence is apparent in the voltage-sensor equipped KCNQ1 channel when assembled with certain accessory subunits (e.g., MiRP1 and 2) [19]. On the opposite end of this spectrum, KcsA channels, now an archetypal model for K^+^-selective pores, appear to exhibit some intrinsic voltage dependence despite lacking a canonical VSD [20],[21]. A second important uncertainty arises in the mechanism of coupling between the voltage sensor and channel pore. In classical models of voltage-dependent gating (such as Shaker or other Kv channels), the VSD strongly influences opening/closing of the pore-forming domain, in the sense that channel open probability (Po) can be reduced to virtually 0 at sufficiently negative voltages and increased to near 1 upon depolarization [22]. In contrast, certain voltage-sensor equipped TRP channels exhibit sustained measurable open probability even at very negative voltages, together with much weaker apparent voltage dependence of gating relative to Kv channels [23]–[25], and incomplete closure can be engineered in classical Kv channels with open state stabilizing mutations at the S6 helix bundle crossing [26]. Such observations indicate that a model of “tight coupling” between the VSD and pore does not apply to all channel types and that the pore domain itself may strongly influence open probability in some ion channels (whether equipped with a voltage sensor or not). In this regard, voltage-sensitive dynamics of the pore-forming module may not always be obvious in ion channels that are strongly governed by motions of the voltage sensor.

Through ongoing characterization of the Kir6.2 channel, we have begun to recognize that substitution of charged residues at pore-lining positions can affect channel gating in very unexpected ways. Kir6.2 is a two transmembrane domain inwardly rectifying K channel, clearly falling into the realm of “voltage sensor-less” ion channels, and assembles with sulfonylurea receptor subunits (SUR1, SUR2A, or SUR2B) to form K_ATP_ channels [27]–[29]. To date, most characterization of K_ATP_ gating has focused on its recognized physiological ligands (notably intracellular nucleotides and anionic phospholipids) [30]–[32]. The present study reveals remarkable voltage-dependent properties that arise in this “voltage-sensor-less” K_ATP_ channel, together with other unrecognized mechanisms of K_ATP_ channel regulation by intracellular ions. We have characterized a mutant Kir6.2 channel that exhibits marked voltage-dependent gating upon membrane depolarization. The voltage dependence of gating of Kir6.2[L157E] is convergent with ligand-dependent gating by ATP and PIP_2_ and is likely to involve the same “gate” as these intrinsic physiological ligands of the K_ATP_ complex. We demonstrate that the voltage- and ligand-dependent gating of these channels is significantly affected by intracellular potassium ions, indicating an interaction between ion permeation and gating and providing a framework for understanding for what is likely to be a general feature of the superfamily of cation channels.

## Results and Discussion

### Voltage-Dependent Activation of the Kir6.2[L157E] Channel

**Figure 1:**
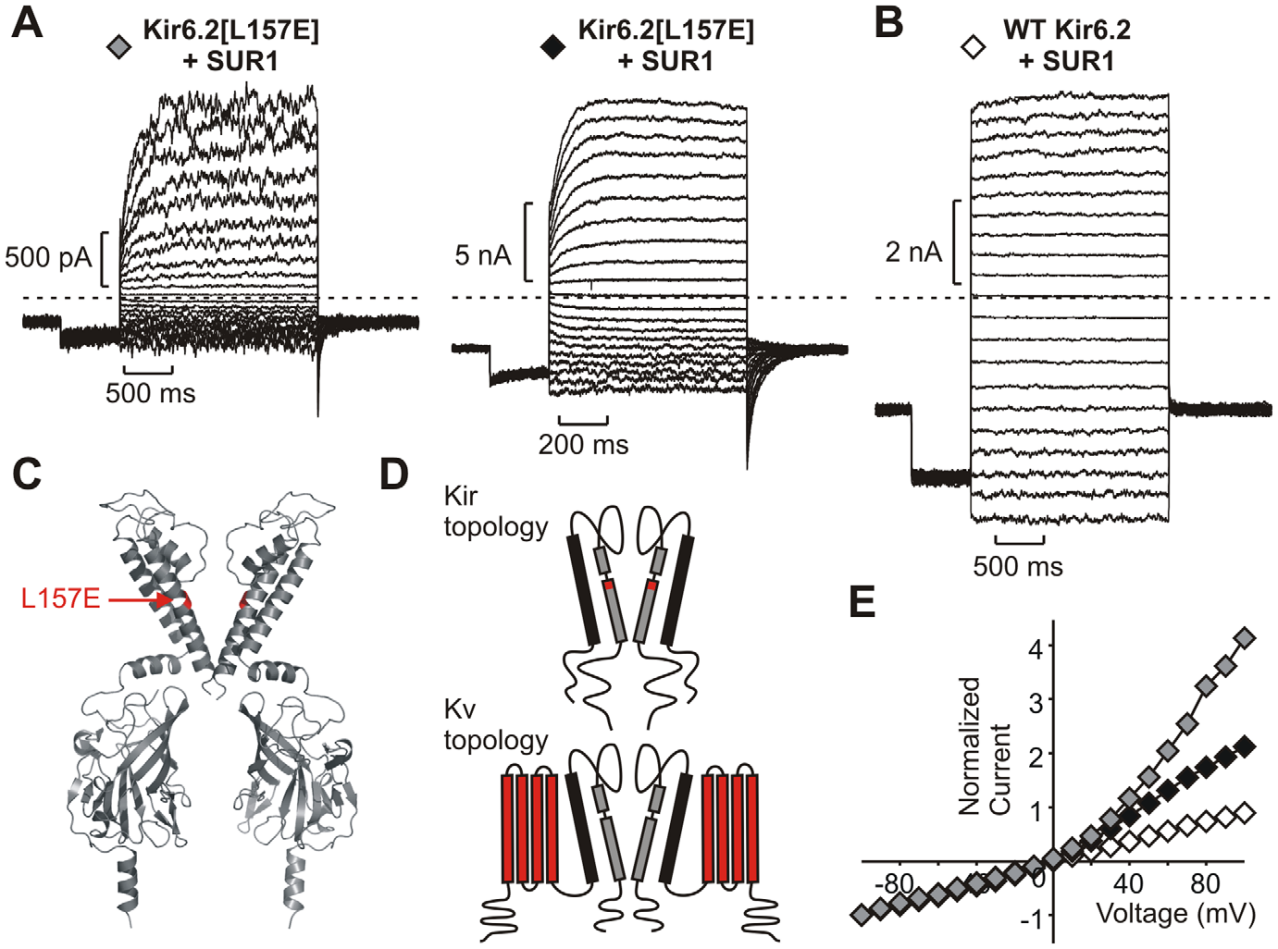
Voltage-dependent activation of Kir6.2[L157E] channels. (A,B) Representative inside-out patch clamp recordings from (A) two different Kir6.2[L157E] membrane patches and (B) a WT Kir6.2 membrane patch (both co-expressed with SUR1). Patches were pulsed to voltages between −100 and +100 mV, with a holding potential of −50 mV. (C) Molecular model of Kir6.2, with residue 157 highlighted in red. (D) Transmembrane topologies of Kir and Kv channel families, with elements underlying voltage-dependent gating colored red in each case. (E) Current-voltage relationships illustrating outward rectification of Kir6.2[L157E] channels. Symbols correspond to the recordings depicted in panels in A–B.

We have characterized the properties of a number of Kir6.2 mutant channels substituted with various charged residues at pore-lining positions. Very unexpectedly, we observed that a single point mutation in the pore-forming subunit of K_ATP_ (Kir6.2[L157E]) generates channels that exhibit voltage-dependent activation (two different patches are depicted in Figure 1A). At negative voltages, patches exhibit a steady-state non-deactivating current. Depolarizing voltage steps result in an instantaneous current jump followed by subsequent activation of current, resulting in an outwardly rectifying current–voltage relationship (Figure 1E). These observations contrast with behavior of WT Kir6.2 channels (Figure 1B), in which significant time-dependent activation is not typical and the macroscopic current-voltage relationship is nearly linear (Figure 1E). Residue 157 is located at a deep pore-lining position in the Kir6.2 inner cavity, directly adjacent to the putative “glycine hinge” (Figure 1C,D). While this single amino acid substitution introduces time-dependent activation somewhat similar to voltage-gated cation channels, the lack of a canonical VSD and a weaker voltage dependence relative to classical Kv channels implies a fundamentally different mechanism is at work (Figure 1D).

The effects of glutamate substitution at residue 157 are position specific. We have examined glutamate substitution at multiple other pore-lining positions in Kir6.2 [33] and found no evidence of similar behavior in 129E, 160E, or 164E channels (see Figure S1). Glutamate substitution at position 168 results in somewhat unusual effects on conduction, including intrinsic inward rectification (in the absence of intracellular blockers), but these do not resemble the unique voltage-dependent activation of Kir6.2[L157E].

### Open Probability Is Voltage Dependent in Kir6.2[L157E]

**Figure 2:**
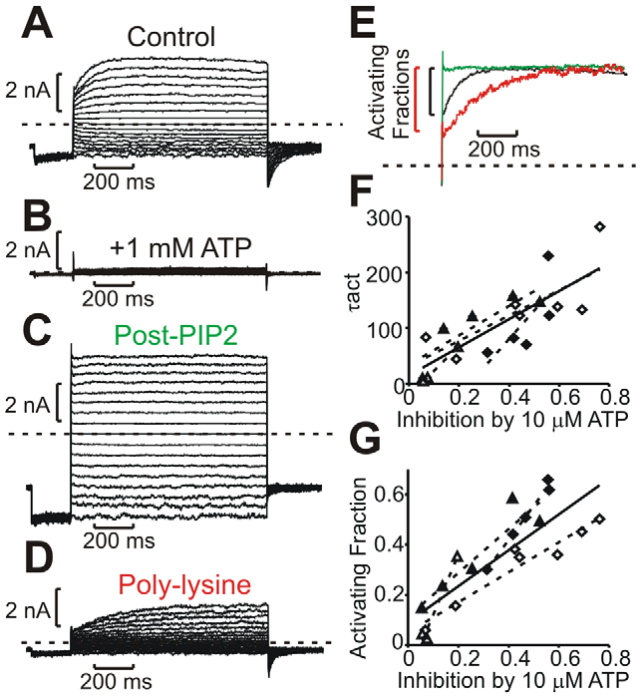
Voltage-dependent gating of Kir6.2[L157E] channels interacts with PIP_2_-regulated open probability. (A–D) Representative current traces from a Kir6.2[L157E] membrane patch, (A) immediately after excision, (B) in 1 mM ATP, (C) after exposure to 5 µg/mL PIP_2_, (D) after brief exposure to the PIP_2_ antagonist poly-lysine. (E) Currents from A, C, and D are normalized to peak to illustrate the effects of basal open probability (determined by PIP_2_) on activation kinetics and on the activating fraction of peak current. (F,G) Compiled data from 4 Kir6.2[L157E] membrane patches, illustrating the relationship between ATP sensitivity (an index of open state stability), and (F) activation kinetics or (G) activating fraction. At higher open state stability, a smaller fraction of the peak current exhibits time-dependent activation, and the kinetics of activation are markedly faster. In (F,G), data are presented from four patches, with each symbol type reflecting a different membrane patch. Dashed lines are linear regression fits to each individual patch, while the solid line is a fit to the entire data set.

Several observations confirm that voltage-dependent activation of Kir6.2[L157E] is due to channel gating, rather than an alternative mechanism such as relief of block, a voltage-dependent change in conductance, or activation of an alternative channel type in the patch. Firstly, we examined the effects of ligands known to alter channel Po in WT Kir6.2 and other Kirs, namely PIP_2_ (which is stimulatory and enhances open state stability/open probability) and poly-lysine (which is inhibitory and reduces open state stability). After inside-out patch excision, voltage-dependent currents were inhibited by internal ATP (Figure 2A,B) indicating that currents were indeed carried by K_ATP_ channels. We subsequently applied either PIP_2_ or poly-lysine to the cytoplasmic face of the membrane and subjected patches to a series of voltage steps. A pattern emerged in which PIP_2_ application resulted in accelerated kinetics of activation and a reduction in the activating fraction of macroscopic currents. This effect could be saturated with sufficient PIP_2_ application, to the point where an activating component of current was no longer apparent (Figure 2C). Application of poly-lysine, which reduces open probability of Kir channels by shielding negatively charged headgroups of anionic phospholipids (e.g., PIP_2_) [34], led to opposite effects: slower activation kinetics and an increased activating fraction of current (Figure 2C,D). To further verify that observed currents are indeed carried by Kir6.2, we exploited the fact that the L157E mutation confers strong spermine sensitivity. Application of spermine to excised membrane patches resulted in complete current inhibition at depolarized voltages (Figure S2), confirming that the observed voltage dependence is intrinsic to these channels and that development of leak does not contribute to current properties observed after subsequent treatment with activating agents such as PIP_2_.

Manipulation of open state stability over a wide range (using PIP_2_ or poly-lysine) illustrates a relationship between open state stability and the properties of voltage-dependent gating, demonstrated in normalized current records (Figure 2E) and in data from multiple patches (Figure 2F,G). Using ATP sensitivity (fractional inhibition in 10 µM ATP) as an index of open state stability [30],[31], there is a clear relationship between open state stability and both the activation time constant and the fractional activating component of macroscopic current. At low open state stability (after poly-lysine, low Po, channels very sensitive to ATP), the activating fraction is large and the activation kinetics are slow (Figure 2E, red trace, and Figure 2F,G). In contrast, at high open state stability (after PIP_2_, high Po, channels weakly sensitive to ATP), the activating component of current decreases, and the activation time constant is accelerated (Figure 2F,G). PIP_2_ exposures sufficient to saturate open probability virtually eliminate voltage-dependent gating (because channels are maximally open at all voltages, Figure 2E, green trace). The demonstrated relationship between channel Po and voltage-dependent gating, and especially the loss of voltage dependence at saturating open probability, indicates that the gating of Kir6.2[L157E] arises primarily from voltage-dependent changes in Po. Also, convergence of the novel voltage-dependent gating mechanism and intrinsic PIP_2_ regulation suggests that voltage is influencing the ligand-operated (ATP/PIP_2_) gate of Kir6.2.

### Multi-Tiered Kinetic Model of Voltage-Dependent Gating

**Figure 3:**
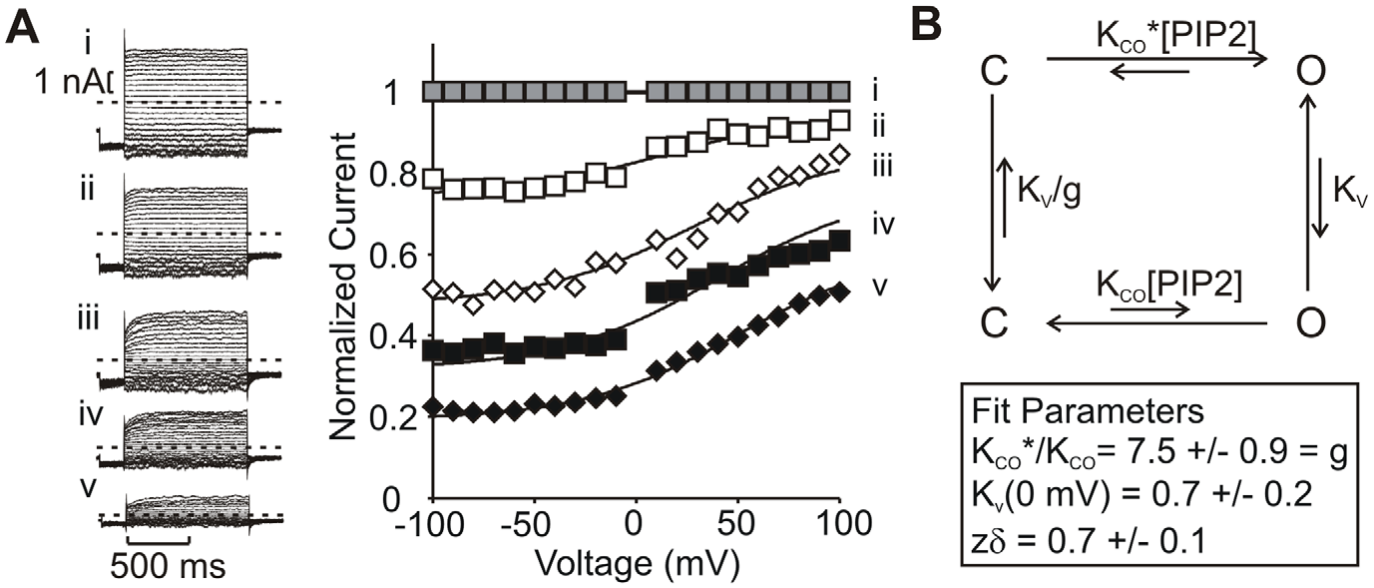
Kinetic model describing voltage-dependent activation of Kir6.2[L157E] over a range of voltage and basal open probability. (A) Current records collected after saturating open probability with PIP_2_ (i), followed by progressive reduction of open state stability with brief applications of poly-lysine (ii–v). In the right-hand panel, steady-state currents were normalized to fully activated currents (record i) to illustrate the extent of activation at each voltage. (B) Kinetic model depicting two tiers of gating—a low Po tier (lower) and a high Po tier (upper). In the high Po tier, the KCO equilibrium constant is 7-fold larger. Equilibria between the high and low Po tiers are governed by the Kv constant, and g is a factor included to preserve reversibility (g = K_CO_*/K_CO_).

It is notable that voltage-dependent properties can vary from patch to patch, as ambient lipid levels (likely PIP_2_) vary (Figure 1A,B, Figure 2A) [35]. The relationship between open state stability and voltage-dependent gating is further illustrated in Figure 3 and provides an additional perspective to the effects described in Figure 2. After excision, open probability was first brought to saturation by application of PIP_2_ [30] (Figure 3A,i), and then iteratively reduced with brief poly-lysine applications (Figure 3A,ii–v). Currents after each poly-lysine exposure were normalized to the “fully activated” currents (condition (i)). Notably, at low open state stability (low PIP_2_ levels, e.g., Figure 3A,v, or Figure 2D), currents at negative voltages are small but can increase several-fold upon depolarization. The result is a large activating fraction of outward current (in normalized traces, Figure 2E), although the absolute currents do not reach the same level as observed in higher PIP_2_ conditions. As open state stability is increased, basal currents at negative voltages are larger, and the fraction of outward current that exhibits time-dependent activation is necessarily smaller. Importantly, the emergence of these patterns are not due to electrostatic effects on permeation, because neither PIP_2_ or poly-lysine affect the Kir6.2 single channel conductance[31],[34].

The steady-state voltage and PIP_2_ dependence of activation of Kir6.2[L157E] can be reasonably well fit over a wide range of open state stability with a simple allosteric model (Figure 3B). The model describes an open-closed equilibrium (K_CO_) governed by the membrane PIP_2_ content, with channels able to occupy two different gating tiers distinguished by the K_CO_ equilibrium constant (the low Po tier has a small K_CO_, and the high Po tier a higher K_CO_—indicated by K_CO_* in Figure 3B). For clarity, the K_CO_·[PIP_2_] term directly reflects what we have referred to as “open state stability” thus far. The partition between high and low Po tiers is described by a voltage-dependent equilibrium constant (K_v_). The model was fit simultaneously to data over a wide range of open state stability (by varying [PIP_2_] in the model). Similar experiments and analysis in four patches indicates a K_v_(0 mV) equilibrium constant of 0.7±0.2 (with an effective valence of 0.7±0.1) and a ∼7-fold stabilization of the K_CO_ equilibrium constant in the high Po tier (K_CO_*/K_CO_ = 7.5±0.9, this is also the value of the reversibility factor g).

Although a potential physical mechanism underlying voltage-dependent activation will be discussed in detail in subsequent sections, two elements of this kinetic model are worth noting. In simple terms, the model implies that the Kir6.2[L157E] channel operates in two gating tiers (high Po and low Po), with the partition between gating tiers influenced by voltage. Secondly, experimental data seem to preclude any simple model in which an open-closed equilibrium is directly controlled by voltage—such models predict that sufficiently high voltages would open channels to a similar level (and sufficiently negative voltages would close channels), irrespective of basal open probability, a prediction that fails to match the observed behavior (Figure 3).

### Single Channel Properties of WT and Kir6.2[L157E] Channels

**Figure 4:**
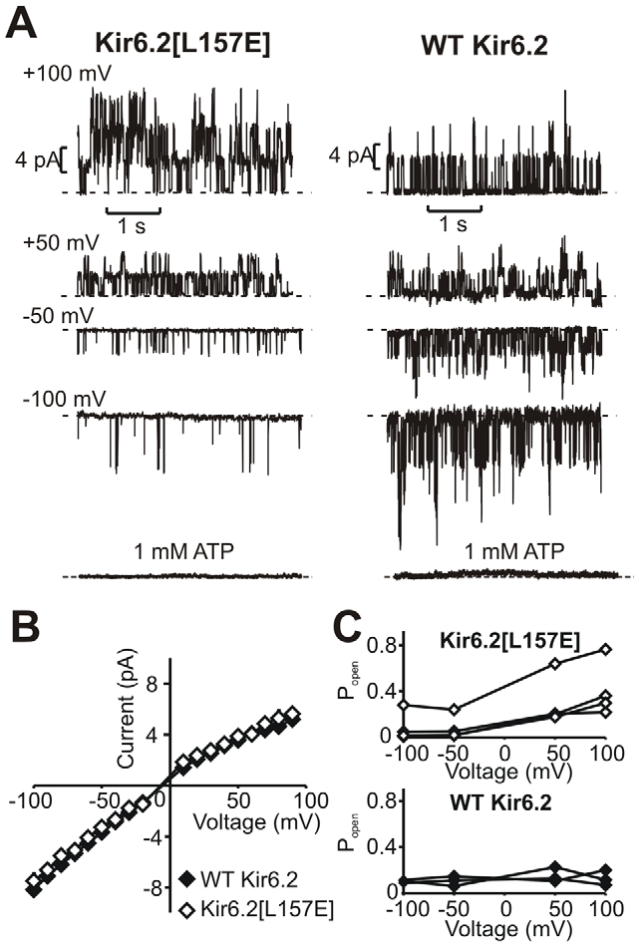
Depolarization increases open probability of Kir6.2[L157E] channels. (A) Current records from membrane patches containing few channels (likely three per patch) for Kir6.2[L157E] or WT Kir6.2 recorded in symmetrical 150 mM K^+^ conditions. (B) Single-channel currents between −100 and +100 mV in WT Kir6.2 and Kir6.2[L157E] channels. The L157E mutation has no significant effect on single channel conductance. (C) Open probability of Kir6.2[L157E] (top) or WT Kir6.2 (bottom) channels measured from membrane patches containing 1−5 channels, between −100 and +100 mV (*n* = 3 for WT and 4 for L157E).

We also measured currents from patches expressing small numbers of channels (1–5 per patch) to determine the effects of voltage on unitary conductance and Po (Figure 4A). WT Kir6.2 and L157E channels exhibit similar single channel current magnitude, indicating that the L157E mutation has little effect on ion permeation (Figure 4B). Consistent with previous reports [36],[37], single channel current-voltage relationships also exhibited mild inward rectification. Notably, L157E (but not WT) channels exhibit obvious increases in open probability at depolarized voltages (Figure 4A,C). Basal open probability tended to be fairly low in both WT and 157E patches, and so significant increases in open probability were frequently observed for L157E (Figure 4C, accounting for the large “activating fraction” observed in macropatch records—Figure 2E, Figure 3A,v).

### Investigating the Voltage-Sensing Mechanism in Kir6.2[L157E]

#### (i) Internal cations affect gating of Kir6.2 channels

**Figure 5:**
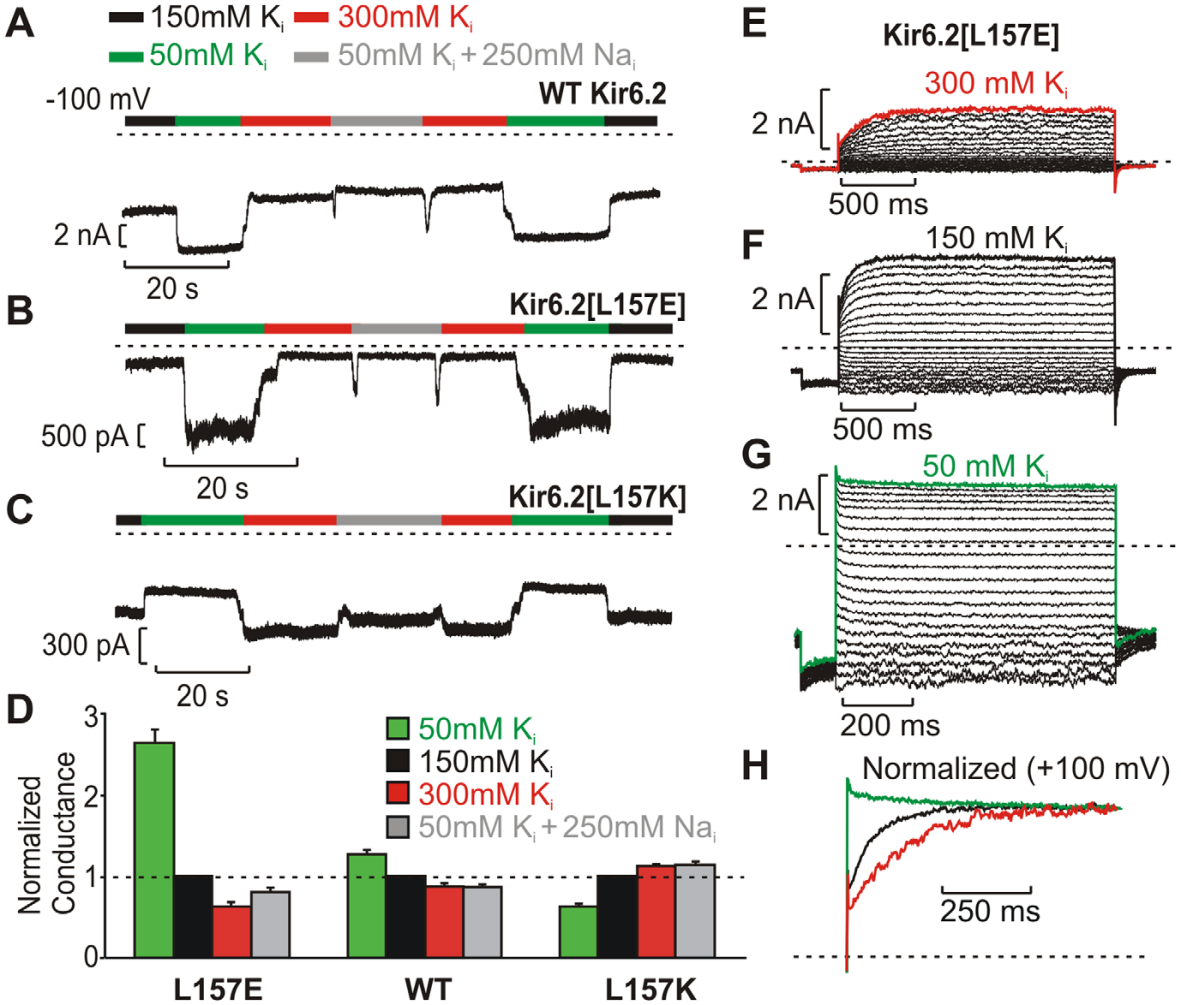
Position 157 affects the internal K^+^ sensitivity of Kir6.2. (A–C) Continuous current records at −100 mV depicting responses to altered internal ionic conditions in inside-out membrane patches expressing (A) WT Kir6.2, (B) Kir6.2[L157E], or (C) Kir6.2[L157K]. The L157E mutation exaggerates the response observed in WT Kir6.2, while the L157K mutation reverses the WT response to intracellular K^+^. (D) Using voltage-step protocols, the chord conductance between −100 and −80 mV was calculated in all K_int_ conditions and normalized to the conductance in 150 mM K^+^ in each patch (*n* = 29 for WT Kir6.2, 19 for Kir6.2[L157E], and 20 for Kir6.2[L157K]). (E–G) Current records from a Kir6.2[L157E] inside-out patch, at voltages from −100 to +100 mV. (H) Currents elicited by a step to +100 mV, normalized to peak current, in the ionic conditions depicted in panels E–G.

In addition to conferring voltage-dependent activation, mutations of Kir6.2 residue 157 surprisingly alter the effects of intracellular K^+^ (K_int_) on channel activity. In both WT Kir6.2 (Figure 5A) and Kir6.2[L157E] (Figure 5B), macroscopic currents (at −100 mV) are reduced in high K_int_ (300 mM), and increased in low K_int_, effects that are substantially enhanced in L157E channels. To account for changes in reversal potential in different K_int_, we used voltage-step protocols (e.g., Figure 5E–G) to calculate the change in macroscopic conductance (chord conductance), by measuring the change in current magnitude between −80 and −100 mV. This demonstrated that conductance of WT patches increased ∼40%, while L157E patches changed 4.5-fold with a switch from 300 mM K_int_ to 50 mM K_int_ (Figure 5D). Normalization to single channel current produced a similar result. This indicates that K^+^ ions significantly influence the gating mechanism in Kir6.2[L157E] channels, with lower K_int_ favoring a higher open probability. Single channel records illustrate dramatic changes in Po as K_int_ is altered (see Figure S3). Together, these observations indicate that intracellular K^+^ ions significantly affect channel open probability in Kir6.2[L157E], and to a lesser degree in WT Kir6.2 channels.

Remarkably, the sensitivity to K_int_ can be reversed by introducing a positive charge at position 157. In Kir6.2[L157K] channels, increasing K_int_ causes an immediate and fully reversible increase of inward currents. This is opposite of what could be accounted for by changes in electrochemical driving force and contrasts dramatically with the effects of K_int_ in WT Kir6.2 and Kir6.2[L157E] channels. It is also notable that these effects are not selective for K^+^. In WT Kir6.2, L157E, and L157K channels, the effects of 300 mM K_int_ are closely mimicked by 50 mM K_int_ supplemented with 250 mM Na_int_ (Figure 5A–D).

Intracellular K^+^ ions also dramatically influence gating kinetics of Kir6.2[L157E] (Figure 5E–H). In 300 mM K_int_, activation kinetics are very slow, and the activating fraction of macroscopic currents is large, resembling the features observed for low Po patches (i.e., following poly-lysine application, Figure 2E). Conversely, currents in 50 mM K_int_ exhibit no time-dependent activation of currents, similar to the behavior of high Po patches (i.e., after saturating PIP_2_ treatment). Overall, these data indicate an especially strong interaction between permeant ions and gating of Kir6.2[L157E] channels.

### 

#### (ii) Voltage-dependent occupancy of the cavity site in K^+^ channels

**Figure 6:**
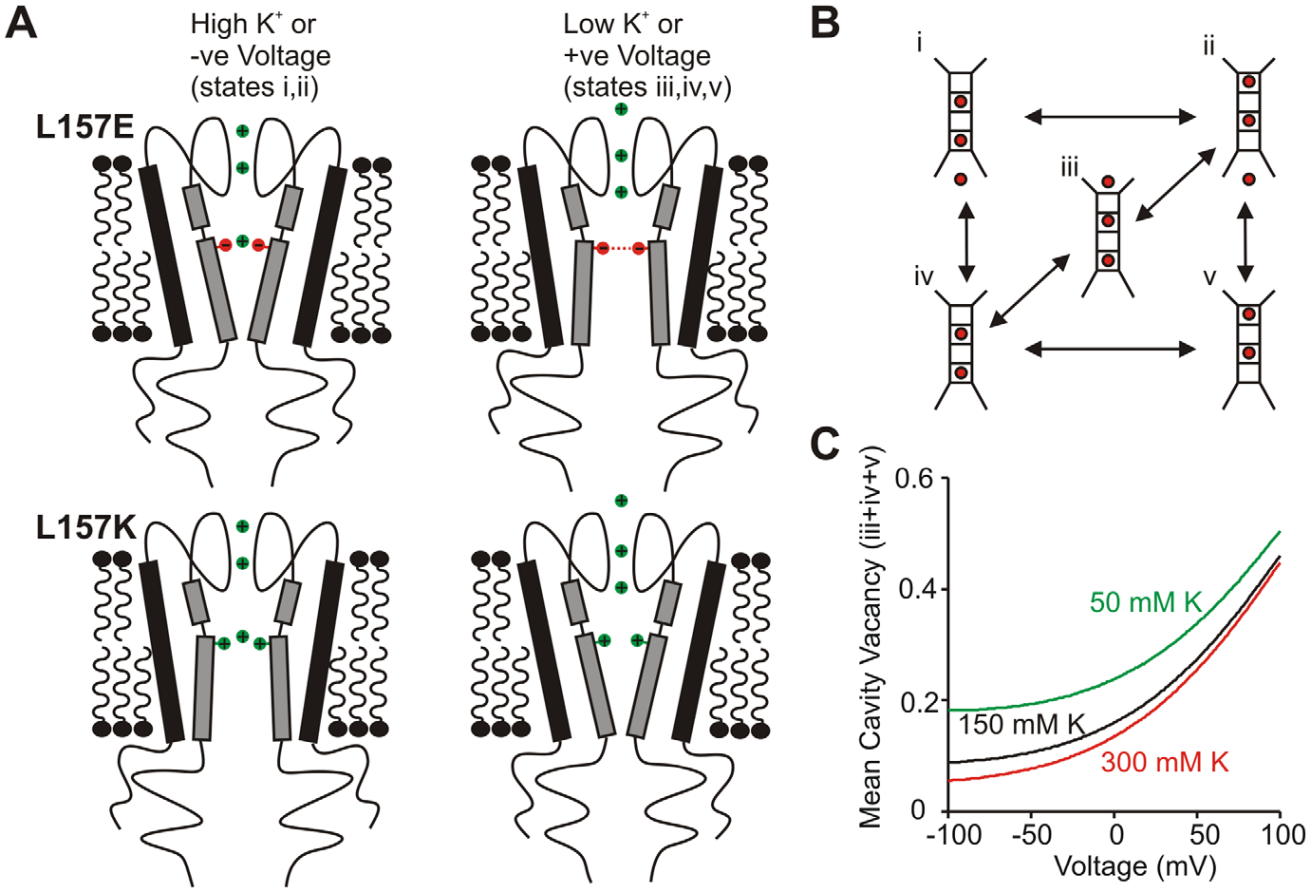
Hypothetical mechanism of convergent regulation by voltage and internal ions. (A) Cation occupancy in the cavity ion binding site will mitigate repulsion between glutamates substituted at position 157, favoring the closed state relative to conditions in which the cavity site is unoccupied. Introduction of a positive charge at position 157 would exhibit an opposite response to cavity site occupancy. (B) Permeation model, with boxes representing selectivity filter binding sites, flanked by an external binding site (top) and the cavity site (bottom). (C) Simulation of voltage and internal K^+^-dependent changes in mean occupancy of the cavity ion binding site (sum of probability of occupancy in states i+ii), using parameters generated to describe permeation through KcsA channels [40].

These observations suggest a “unifying” explanation for the unique behavior of Kir6.2[L157E] channels. Rather than acting as a sensor for changes in transmembrane voltage, we suggest that the L157E mutation generates an environment in which open state stability depends especially strongly on ion occupancy of the inner cavity (Figure 6). Intuitively, this is a straightforward idea: in the absence of a cation in the cavity ion binding site, repulsion between negatively charged side chains would drive the M2 helices apart, favoring channel opening at the helix bundle crossing [38],[39]. Occupancy of the cavity ion site would mitigate this repulsion—157E carboxylates could approach more closely to the central axis of the pore, stabilizing channel closure relative to the unoccupied state. Consistent with our findings, these effects should be reversed with introduction of a positively charged sidechain at position 157 (e.g., L157K, Figure 5C,D). In general terms, the position specificity of the 157E effects (Figure S1) also seems well explained by this idea, because position 157 directly faces the cavity ion binding site and is adjacent to the putative “gating hinge” at glycine G156. Thus, even small motions in this region, perhaps driven by coulombic interactions between neighboring side chains and occupant ions, could be translated into significantly larger motions at the helix bundle crossing.

Such a mechanism may also account for the voltage-dependent activation of Kir6.2[L157E]. Specifically, kinetic models of ion permeation can predict substantial voltage-dependent changes in occupancy of selectivity filter sites and the cavity ion binding site. We have simulated voltage-dependent occupancy of the cavity ion binding site, based on published parameters for a model describing K^+^ permeation in KcsA. (Figure 6B,C) [40]. At *positive* voltages (or low K_int_), the model predicts that occupancy of the cavity ion binding site is low, because the voltage dependence of entry of the cavity ion into the selectivity filter is larger than the voltage dependence for “refilling” this site with an ion from the intracellular solution (Figure 6C). In this way, the gating effects (high Po) observed in low K_int_ can also be achieved at depolarized voltages. At *negative* voltages (or higher K_int_), inward currents saturate the cavity ion binding site. Voltage-dependent cavity site occupancy can be predicted by permeation models in which the movement of the cavity ion into the selectivity filter is more voltage dependent than “refilling” of the cavity site with an intracellular ion and is consistent with the marked asymmetries in the characteristics of single channel openings carrying inward versus outward currents (see Figure S3). Given the generally accepted view that the membrane field is dissipated primarily across the selectivity filter [41],[42], this seems a reasonable assumption. Also, the weak voltage dependence of cavity site occupancy over the experimental voltage range is comparable to the voltage dependence of channel activity (Figure 4).

### Predictions of Permeation-Coupled Gating

**Figure 7:**
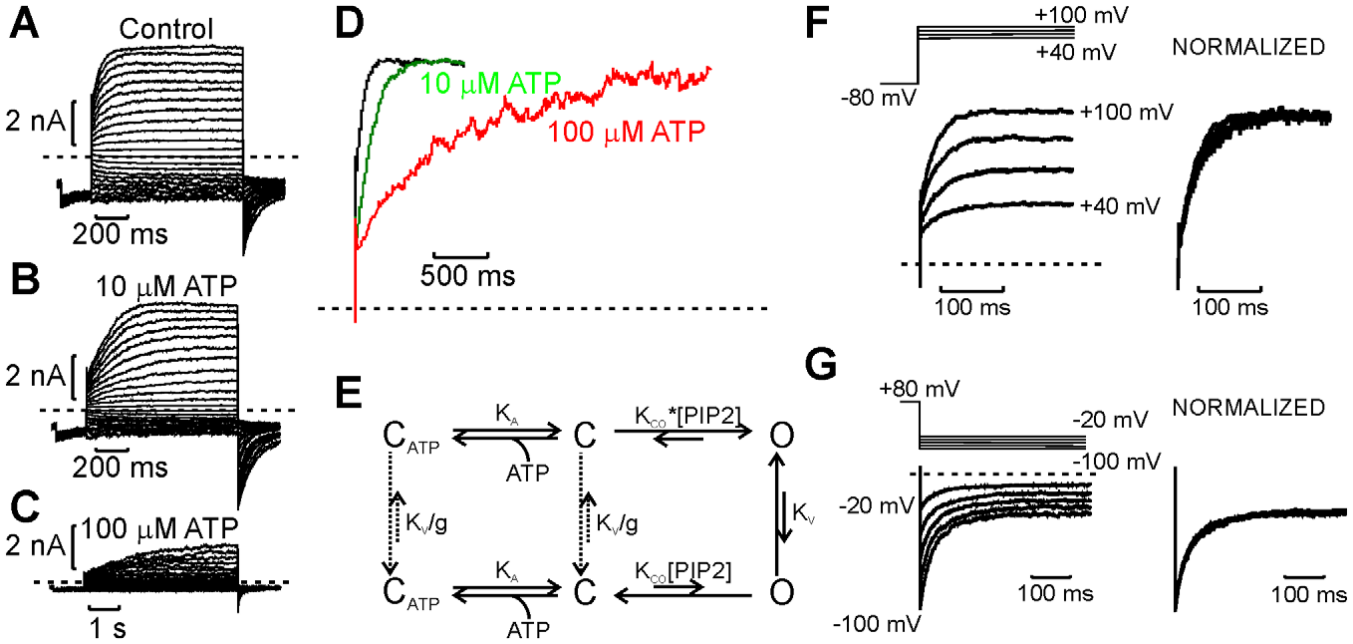
Predictions of gating coupled to changes in permeant ion occupancy. (A–C) In the presence of internal ATP, which acts by prolonging interburst intervals, the kinetics of channel activation are markedly slowed. (D) Normalized current traces recorded at +100 mV, in control and various internal ATP concentrations. (E) Extension of the scheme in Figure 3B, including ATP binding to closed states. In this scheme, ATP stabilizes the channel closed state, thereby prolonging the interburst intervals. (F,G) Kinetics of activation (F) and deactivation (G) and normalized in right-hand panels to illustrate very weak voltage dependence of kinetics.

An important concept of this model is that voltage does not directly drive the channel to open. Rather, channels open stochastically, and rearrangement of ion occupancy after channel opening governs the partition between high and low Po gating modes/tiers. Thus, if anything, the permeant ions themselves can be considered the “voltage sensors.” If this represents the predominant sequence of events during voltage-dependent activation, then the observed gating kinetics should depend primarily on intrinsic channel opening and closing rates (rather than the voltage-driven rate), and ATP stabilization of the closed state (prolongation of single channel interburst intervals) should affect the kinetics of channel opening. This behavior is indeed observed, and the effects can be quite dramatic (Figure 7A–E). Activation kinetics are slowed significantly in 10 µM ATP (Figure 7B,D). In some patches with sufficient current expression and appropriate open state stability, extremely slow activation was also observed in 100 µM ATP (Figure 7C,D). This reflects the infrequency of opening in 100 µM ATP—since openings occur rarely, channels will enter the high Po tier very slowly. These effects can be rationalized by an extension of the simple allosteric model presented earlier (Figure 7E), with the addition of ATP-bound closed states reflecting stabilization of channel closure by ATP. This scheme is not intended to provide a complete description of ATP binding to K_ATP_ channels (see [43]–[45]). However, the model describes the important counter-regulation of K_ATP_ channels by ATP and PIP_2_ [30],[31] and predicts longer channel closures in the presence of ATP. In addition, channel opening upon depolarization and closure after hyperpolarization exhibit very weak voltage dependence (z_act_ = 0.11±0.01, z_deact_ <0.01, Figure 7F,G). Again, this likely reflects the idea that the activation/deactivation kinetics are limited by the intrinsic bursting kinetics of the channel and that voltage is not driving the conformational changes that mediate gating.

### Other Possible Effects of Internal Cations

**Figure 8:**
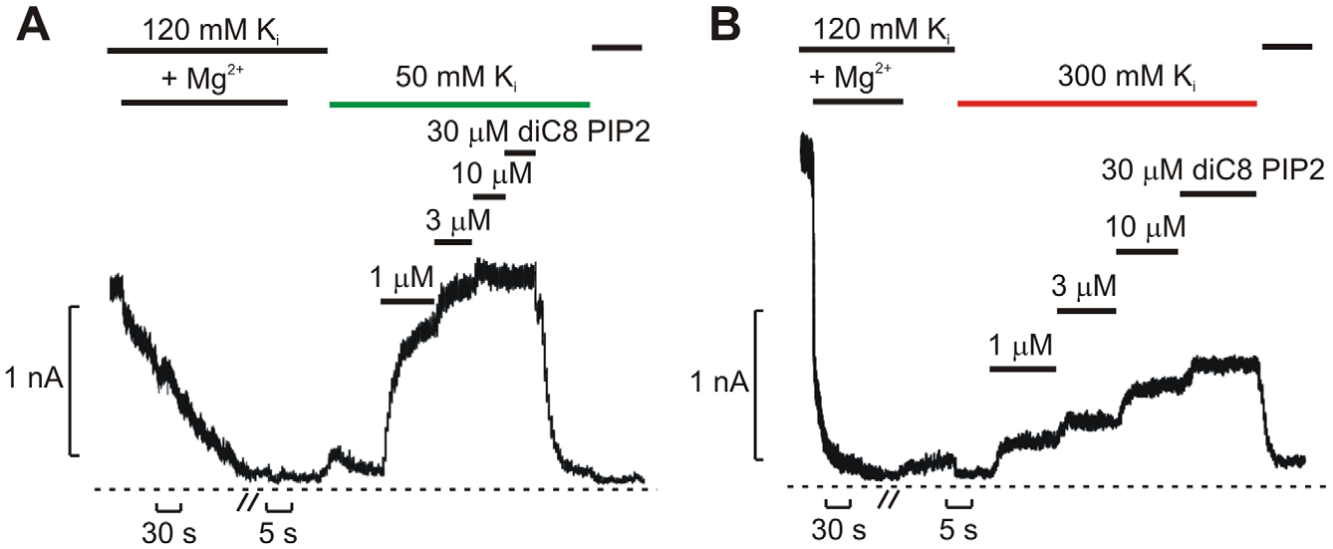
Ionic strength affects channel interactions with PIP_2_. WT Kir6.2 channels were fully run-down in high Mg^2+^ and subjected to increasing concentrations of di-C8 PIP_2_ in either (A) 50 mM or (B) 300 mM internal K^+^. PIP_2_ results in more significant current recovery in low ionic strength conditions. Similar observations were made in six membrane patches.

We have also considered whether intracellular ions might affect channel activity by other mechanisms. We speculated that intracellular ionic strength might affect channel interactions with PIP_2_, and this appears to be a definite possibility. To examine PIP_2_ interactions, WT Kir6.2 channel open probability was “rundown” with a high concentration of Mg^2+^, and then exposed to various concentrations of diC8-PIP_2_, in both high and low ionic strength conditions (Figure 8). It is clear that in low ionic strength, channels are activated more completely and at lower diC8-PIP_2_ concentrations. It appears that ionic strength can indeed alter channel-PIP_2_ interactions, although it should be recognized that this experiment does not establish whether this is a direct effect of ionic strength, or an allosteric effect arising from the actions of ions within the pore (i.e., PIP_2_ interacts with higher affinity with open channels, and so pore-mediated effects of ions on open probability could indirectly affect channel-PIP_2_ interaction). Importantly, it seems unlikely that channel-PIP_2_ interactions would be altered by mutations deep in the inner cavity—and thus the distinct properties of 157K versus 157E cannot be accounted for by this phenomenon. Nevertheless, there is a possibility that intracellular ionic strength affects K_ATP_ channel activity by multiple mechanisms.

### Integrated Voltage- and Ligand-Gating

The K_ATP_ complex is a ligand-gated ion channel, in which diverse cytoplasmic ligands (most notably ATP, ADP, and PIP_2_) determine open probability [30]–[32],[46]. Nucleotide gating is a unique feature of the Kir6 subfamily, but PIP_2_ dependence is common to all members [47]. In the present study, we have uncovered an additional dependence on intracellular cations that confers substantial voltage dependence. Changes in membrane voltage markedly alter the open probability of Kir6.2[L157E] channels, as confirmed by single channel and macroscopic current recordings. Saturation of Po by PIP_2_ (Figures 2, 3) abolishes voltage-dependent activation, confirming that activation reflects increased channel Po. Strong voltage-dependent gating in the absence of a canonical VSD was unpredicted and is remarkable in at least two respects. Firstly, it demonstrates a mechanism by which permeating ions can influence the gating state of the pore-forming module. Secondly, it is imposed on the intrinsic ligand-dependent gating: the kinetic properties and extent of voltage-dependent activation clearly depend on PIP_2_ (Figures 2, 3) and ATP levels (Figure 7), indicating that voltage is influencing the stability of the native PIP_2_/ATP-operated gate.

Converging lines of functional and crystallographic evidence suggest that ligand gating of Kir channels results from closure at or near the inner helix bundle crossing, as it does in Kv channels (see Text S1 for a detailed discussion of this point) [1],[38],[41],[48]–[51]. Our data set is consistent with this model—permeant ions play an important role, but there is no ionic selectivity to the effect, and the critical residue (157) is located in the M2 helix, rather than in the selectivity filter. The voltage-dependent activation of Kir6.2[L157E] likely arises from a state preference for one orientation of permeating ions over another (specifically, whether the cavity site is occupied is vacant). Voltage-dependent ion occupancy, as modeled here (Figure 6), has been inferred from studies of voltage-dependent relief of TEA block in KcsA channels, in which TEA and K^+^ interactions have been hypothesized to depend on voltage-dependent changes in ion occupancy profiles [40],[52]. Although specific interactions with channel gating remain unexamined in KcsA and other channels, it is noteworthy that general features for this mechanism (the K^+^ channel pore module, with a cavity ion binding site) are likely present in all K^+^ channels, and the general principles could extend to other channel types irrespective of structure/sequence.

In Kv channels, Po is strongly controlled by the canonical VSD [22]. However, various channel types exhibit considerable diversity in the apparent strength of coupling between the voltage sensor and pore. As alluded to in the introduction, there is growing recognition of nominally “voltage-gated” channels that show far weaker voltage dependence than close *Shaker* homologues and exhibit persistent open probability at negative voltages [25]. Such features may indicate that coupling between the voltage sensor and pore is relatively weak and that the pore-forming module can significantly affect open state stability/open probability—indeed mutations in the helix bundle crossing region can result in persistent opening of Kv channels [26]. Furthermore, many voltage-gated channel assemblies, perhaps most notably the KCNQ1/KCNE1 complexes, exhibit activation kinetics that appear to be considerably slower than the kinetics of voltage-sensor equilibration [53]. Similarly, a small voltage dependence is generally attributed to the final concerted opening step of the pore module in widely studied channels like *Shaker* and BK [22],[54], although the mechanism for this voltage dependence is not well understood. Growing recognition of diverse non-canonical mechanisms of voltage sensing, in KcsA [20],[21], in the present study, and in a recent report of introduced voltage dependence in CNG channels [55], suggest important avenues to investigate the role of the pore-forming module in controlling open probability.

Finally, while Kir6.2[L157E] exhibits an obvious voltage-dependent phenotype, the presence of a negatively charged side chain may not be an absolute requirement, since the same underlying feature is weakly detectable in WT channels. A small hint of this phenomenon is apparent in Figure 1C, and we have included a more marked example in Figure S4. While not as dramatic as the voltage-dependent activation of Kir6.2[L157E], these features can be quite obvious and are exaggerated in modest inhibitory concentrations of ATP. These observations suggest that other features (beyond electrostatic interactions of charged side chains and the cavity ion) can generate some state preference for specific configurations of permeant ions. One potential candidate in K^+^ channels is stabilization of the cavity ion by the pore helices, which may be more prominent in the closed versus open state [56], and thus might underlie some energetic preference for one configuration of permeant ions over another in different channel states.

### Conclusions

We have characterized a unique and unexpected voltage-dependent activation feature of a ligand-gated Kir channel. The voltage dependence arises from voltage-dependent interactions of permeating ions with the same gate as that controlled by gating ligands, providing a unifying interaction between two fundamental processes of gating. The effects of the pore-forming module in regulating the kinetics and properties of voltage-dependent gating tend to be overlooked, since voltage dependence of cation channels is generally attributed to motions of a canonical VSD. However, particularly in cation channels that exhibit relatively weak voltage dependence and persistent conductance at negative voltages, we suggest that the pore-forming module itself may be an important structural element in the regulation of voltage dependence and kinetics of channel gating.

## Materials and Methods

### Expression of K_ATP_ Channels in COSm6 Cells

Point mutations were prepared using the Stratagene Quickchange kit, on a background of WT mouse Kir6.2. COSm6 cells were transfected with pCMV6b-Kir6.2 (with mutations as described), pECE-SUR1, and pGFP using the Fugene 6 transfection reagent. Patch-clamp experiments were made at room temperature, using a chamber that allowed rapid solution exchange, or the Dynaflow capillary chip-based platform (Cellectricon Inc.), with DF-16 Pro II chips [57].

Data were typically filtered at 1 kHz, and signals were digitized at 5 kHz and stored directly on computer hard drive using Clampex software (Axon Inc.). The standard pipette (extracellular) and bath (cytoplasmic) solution used in these experiments had the following composition: 140 mM KCl, 1 mM K-EGTA, 1 mM K-EDTA, 4 mM K_2_HPO_4_, pH 7.3. For 50 mM K_int_, 300 mM K_int_, and 50 mM K_int_ + 250 mM Na_int_ solutions, all buffer components were kept at the same concentration, with changes only to the indicated principal solutes (KCl or NaCl). Chemicals were all purchased from Sigma-Aldrich, or FLUKA, with the exception of PIP_2_ (phosphatidylinositol 4,5-bisphosphate, Avanti).

### Kinetic Modeling

Models describing steady-state voltage dependence of activation, and ion occupancy, were generated using the “Q-matrix method” [58]. Matrix Q was constructed such that each element (i,j) was equal to the rate constant from state i to state j, and each element (i,i) was set to be equal to the negative sum of all other elements in row i. State occupancy at time t was calculated as p(t) = p(0)e^Qt^, where p(t) is a row vector containing elements corresponding to occupancy of each state in the model at time t. All tasks required for solving these equations were performed in MathCad 2000. Parameters describing ion occupancy are replicated from an earlier published model describing ion permeation through KcsA channels, with the exception of a repulsion factor describing the interaction of ions in adjacent binding sites [40]. We reduced the published repulsion factor for the simulations described in Figure 6C, as we found this predicted higher cavity occupancy at extreme negative voltages.

## Supporting Information

Figure S1 **Voltage-dependent activation of L157E is position specific.** (A) Pore-lining positions substituted with glutamate are highlighted in a molecular model of the Kir6.2 inner cavity. Position 157 is highlighted in red. (B-F) Currents elicited from inside-out membrane patches expressing each glutamate mutant. Pronounced voltage-dependent activation is only observed in Kir6.2[L157E] channels.
(0.16 MB TIF)

Figure S2 **Polyamine block of Kir6.2[L157E] channels.** Representative records depict voltage-dependent activation of Kir6.2[L157E] channels immediately after inside-out patch excision (A). In (B), voltage-dependent activation is abolished after saturation of open probability by application of PIP_2_. (C) Complete and steeply voltage-dependent inhibition of outward currents in 100 mM spermine indicates that currents are carried through Kir6.2[L157E] and that effects of PIP_2_ are not due to a non-specific leak or activation of another channel subtype.
(0.01 MB TIF)

Figure S3 **Potassium and voltage-dependent effects on unitary currents in Kir6.2[L157E] channels and WT Kir6.2 channels.** (A,B,C) Single channel records at −90 mV (left) and +90 mV, in various Ki conditions, as indicated. The properties of single channel currents vary dramatically between positive and negative voltages. At positive voltages, extremely long uninterrupted openings are apparent, whereas more frequent flicker-like closures are observed at negative voltages. Additionally, intracellular K+ dramatically affects open probability, with high Ki reducing channel Po. (D) Similar features are also observed in WT Kir6.2 channels, with long openings observed at depolarized voltages and far more frequent flickering closures at negative voltages. These marked asymmetries in the characteristics of single channel openings carrying inward versus outward currents may not be entirely surprising (given the asymmetric structure of an ion channel). These are included as somewhat anecdotal support for microscopic features of gating and permeation changing significantly with voltage. This asymmetry is especially apparent in low K_int_ conditions (Figure S3A), where negative voltages are characterized by rapid flicker-like closures, while no closures are observed at positive voltages.
(0.06 MB TIF)

Figure S4 **Voltage-dependent activation of WT Kir6.2 channels.** Inside-out patch-clamp recordings of WT Kir6.2 in symmetrical K^+^ concentrations, in the presence or absence of 10 µM ATP. We frequently observed modest activation of WT Kir6.2 at depolarized voltages (A). Though not as pronounced as in Kir6.2[L157E] channels, it is quite apparent and becomes more obvious in modest ATP concentrations (B). It is possible that experimental conditions can be devised to maximize this voltage dependence of WT Kir6.2.
(0.02 MB TIF)

Text S1 **Where is the ligand/voltage-sensitive gate located?** Supplemental text presents evidence related to the localization of the ATP/PIP2-operated gate in Kir6.2 channels and ligand-operated gating in other Kir channels.
(0.06 MB DOC)

## Abbreviations

BK: large conductance potassium channel
CNG: cyclic nucleotide gated channel
KATP: ATP-sensitive potassium channel
Kint: internal potassium concentration
Kir: inwardly-rectifying potassium channel
Kv: voltage-gated potassium channel
MiRP: MinK-related peptide
PIP2: phosphatidyl inositol bis-phosphate
Po: open probability
SUR: sulfonylurea receptor
VSD: voltage-sensing domain
