# Supplementary figures and images for: Voltage-Dependent Gating in a “Voltage Sensor-Less” Ion Channel
Source: PLoS Biol. 2010 Feb 23;8(2):e1000315. doi: 10.1371/journal.pbio.1000315 (PMC2826373; doi:10.1371/journal.pbio.1000315)

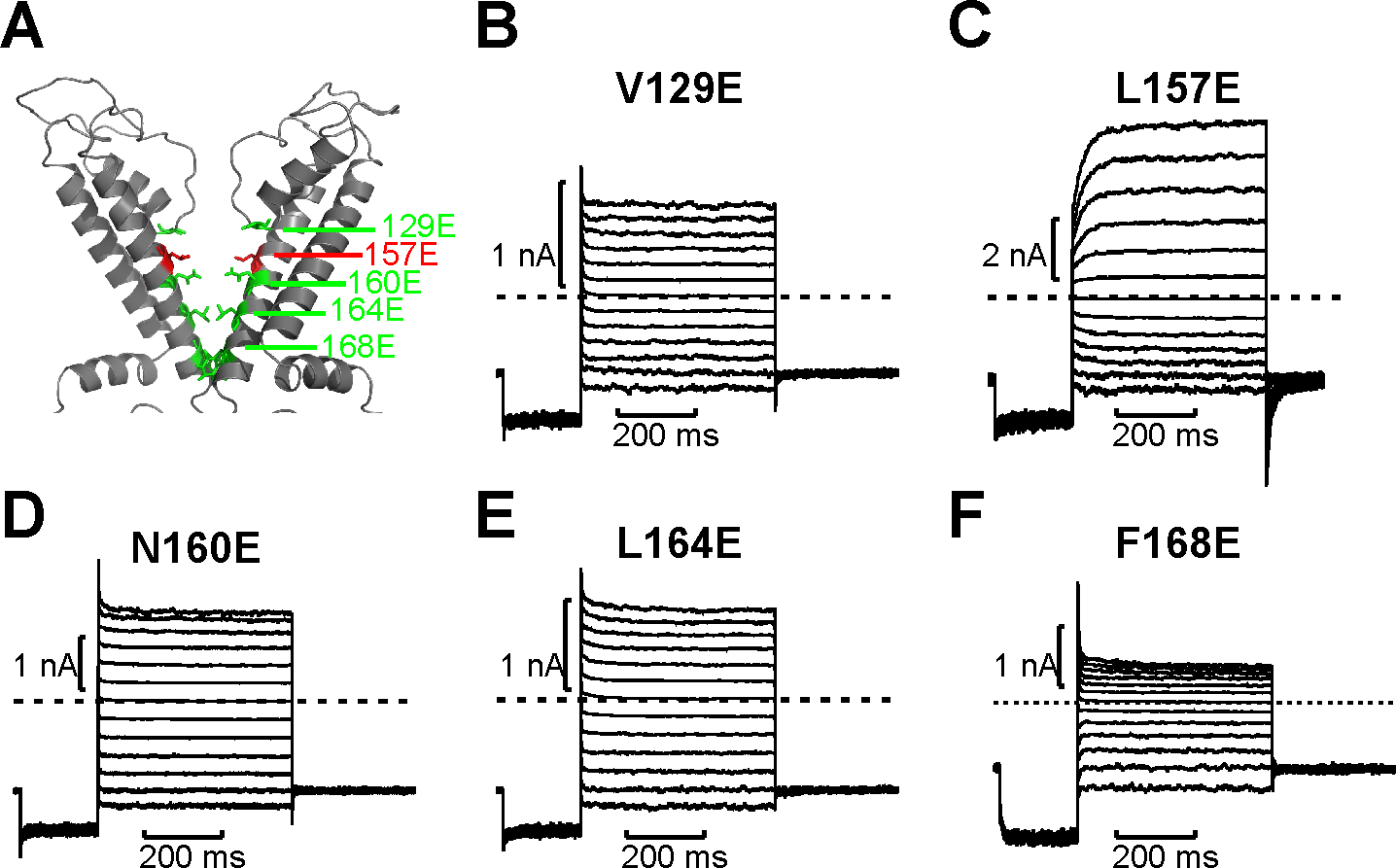

Supplement: Figure S1 — Voltage-dependent activation of L157E is position specific. (A) Pore-lining positions substituted with glutamate are highlighted in a molecular model of the Kir6.2 inner cavity. Position 157 is highlighted in red. (B-F) Currents elicited from inside-out membrane patches expressing each glutamate mutant. Pronounced voltage-dependent activation is only observed in Kir6.2[L157E] channels. (0.16 MB TIF) [file pbio.1000315.s001.tif]
