# Supplementary material for: Voltage-Dependent Gating in a “Voltage Sensor-Less” Ion Channel
Source: PLoS Biol. 2010 Feb 23;8(2):e1000315. doi: 10.1371/journal.pbio.1000315 (PMC2826373; doi:10.1371/journal.pbio.1000315)
Supplement: Text S1 — Where is the ligand/voltage-sensitive gate located? Supplemental text presents evidence related to the localization of the ATP/PIP2-operated gate in Kir6.2 channels and ligand-operated gating in other Kir channels. (0.06 MB DOC) [file pbio.1000315.s005.doc]

**TEXT S1**

**Where is the ligand/voltage-sensitive gate located?**

Although permeant ions have a strong influence over the observed voltage-dependent gating mechanism, the results are most consistent with a model of gating by the helix bundle crossing. Notably, there is no obvious K-selectivity to the voltage-dependent effect (Fig. 5A-D). In addition, the observed ion concentration-dependence is inconsistent with well-described mechanisms of selectivity filter gating (eg. C-type inactivation and related processes), in which collapse/closure of the filter is favored by depletion of ions. The exaggerated voltage-dependence in L157E influences the intrinsic (ATP/PIP_2_) ligand-dependent gating mechanism (Figs. 2,3,7), and multiple consistent lines of functional and crystallographic evidence indicate that ligand-gating of Kir channels results from closure at or near the inner helix bundle crossing, as it does in Kv channels [1,2,3 ,4–7]. Collectively, these observations suggest a mechanism that differs significantly from the voltage-dependent C-type inactivation process mediated by the KcsA selectivity filter. One study in Kir6.2 has suggested a ligand-dependent gating mechanism mediated by the selectivity filter [8], but the balance of evidence favors a mechanism involving the helix bundle crossing. To consider this question in more detail, multiple experimental findings converge to make a strong case for a model of M2 helix movement:

(1) The strong effects of residue 157 (in M2) on voltage-dependent gating and Kint-dependence, the primary experimental finding in our study. This residue is located in the inner cavity and does not invoke gating at the selectivity filter.

(2) The lack of any K+ selectivity of the observed effects. That Na^+^ can be substituted for K^+^ with virtually identical effects on channel activity (Fig. 5) suggests that the permeant ion effect is mediated by an ion in the inner cavity, and not by an ion in the selectivity filter.

(3) The lack of consistency between the permeant ion dependence and established mechanism(s) of selectivity filter gating. Established mechanisms of gating by the selectivity filter involve inactivation related to depletion of ions in the filter [9], typically favoring a non-conducting state in low ionic strength, and a conducting state in high ionic strength (especially dependent on extracellular ions). However, we observe the opposite – high ionic strength dramatically reduces channel open probability, low ionic strength increases open probability, and intracellular ions are the strongest determinant of open probability. Voltage-dependent gating of the selectivity filter, as reported in KcsA, appears to be a ‘special case’ of C-type inactivation, in which a specific residue behind the selectivity filter undergoes a voltage-dependent rearrangement that appears to be independent of the KcsA ligand-operated (pH) gate [10]. Macroscopic currents in WT Kir6.2 are substantially different, because they exhibit very little voltage-dependence, indicating a weak contribution of voltage-dependent rearrangements of the selectivity filter (or other structural motifs). It seems unlikely that an amino acid substitution outside the selectivity filter, with little or no effect on channel conductance, would introduce propensity for voltage-dependent rearrangement of the selectivity filter. In addition, as we discuss below, the voltage-dependent gate in L157E channels is the same as the ligand operated-gate – an important difference from KcsA.

(4) The voltage-dependence introduced by L157E is acting through the intrinsic (ATP/PIP2) ligand–operated gate (Figs. 2,3,7,S2), and significant diverse evidence points to the helix bundle crossing as the gating site for ligand-gating in KATP and other Kir channels. Multiple studies clearly indicate that ATP-dependent closure restricts access of cysteine reactive probes to the inner cavity region [2], and traps polyamine blockers within the inner cavity of Kir6.2 [11]. In addition, ATP inhibition slows the rate of Cd^2+^ modification of Kir6.2 inner cavity cysteines [12]. Mutations in the bundle crossing region also appear to impair ATP-dependent inhibition of Kir6.2 [13]. There is other important data in this regard. Multiple crystal structures of KirBac [7] or chimeric Kir channels [6] exhibit a tight constriction of an aromatic residue at the helix bundle crossing (corresponding to F168 in Kir6.2). EM structures of KirBac3.1 in multiple configurations also suggest Kir channel conformational changes in the bundle crossing region [14]. In sum, the notion that ATP-mediated gating of Kir6.2 involves the helix bundle crossing is a plausible and well-supported model of conformational changes associated with ATP/PIP2-mediated gating.

**References**

1. Long SB, Campbell EB, MacKinnon R (2005) Voltage sensor of Kv1.2: structural basis of electromechanical coupling. Science 309: 903-908.

2. Phillips LR, Enkvetchakul D, Nichols CG (2003) Gating dependence of inner pore access in inward rectifier K(+) channels. Neuron 37: 953-962.

3. Jiang Y, Lee A, Chen J, Cadene M, Chait BT, et al. (2002) The open pore conformation of potassium channels. Nature 417: 523-526.

4. Armstrong CM (1971) Interaction of tetraethylammonium ion derivatives with the potassium channels of giant axons. J Gen Physiol 58: 413-437.

5. Del Camino D, Yellen G (2001) Tight steric closure at the intracellular activation gate of a voltage-gated K(+) channel. Neuron 32: 649-656.

6. Nishida M, Cadene M, Chait BT, MacKinnon R (2007) Crystal structure of a Kir3.1-prokaryotic Kir channel chimera. EMBO J 26: 4005-4015.

7. Kuo A, Gulbis JM, Antcliff JF, Rahman T, Lowe ED, et al. (2003) Crystal structure of the potassium channel KirBac1.1 in the closed state. Science 300: 1922-1926.

8. Proks P, Antcliff JF, Ashcroft FM (2003) The ligand-sensitive gate of a potassium channel lies close to the selectivity filter. EMBO Rep 4: 70-75.

9. Baukrowitz T, Yellen G (1996) Use-dependent blockers and exit rate of the last ion from the multi-ion pore of a K+ channel. Science 271: 653-656.

10. Cordero-Morales JF, Cuello LG, Perozo E (2006) Voltage-dependent gating at the KcsA selectivity filter. Nat Struct Mol Biol 13: 319-322.

11. Phillips LR, Nichols CG (2003) Ligand-induced closure of inward rectifier Kir6.2 channels traps spermine in the pore. J Gen Physiol 122: 795-804.

12. Loussouarn G, Makhina EN, Rose T, Nichols CG (2000) Structure and dynamics of the pore of inwardly rectifying K(ATP) channels. J Biol Chem 275: 1137-1144.

13. Rojas A, Wu J, Wang R, Jiang C (2007) Gating of the ATP-sensitive K+ channel by a pore-lining phenylalanine residue. Biochim Biophys Acta 1768: 39-51.

14. Kuo A, Domene C, Johnson LN, Doyle DA, Venien-Bryan C (2005) Two different conformational states of the KirBac3.1 potassium channel revealed by electron crystallography. Structure 13: 1463-1472.
